# Information conveyed by electrical diaphragmatic activity during unstressed, stressed and assisted spontaneous breathing: a physiological study

**DOI:** 10.1186/s13613-019-0564-1

**Published:** 2019-08-14

**Authors:** Lise Piquilloud, François Beloncle, Jean-Christophe M. Richard, Jordi Mancebo, Alain Mercat, Laurent Brochard

**Affiliations:** 1 Medical Intensive Care Department, University Hospital of Angers, University of Angers, 4, Rue Larrey, 49100 Angers, France; 2 Adult Intensive Care and Burn Unit, University Hospital and University of Lausanne, Rue du Bugnon 46, 1011 Lausanne, Switzerland; 3 SAMU74, Emergency Department, General Hospital of Annecy, 1, Av de l’hôpital, 74370 Epagny Metz-Tessy, France; 4 INSERM, UMR 955, Créteil, France; 5 Intensive Care Department, Sant Pau Hospital, Carrer de Sant Quinti 89, 08041 Barcelona, Spain; 6 Interdepartmental Division of Critical Care Medicine, University of Toronto, Toronto, Canada; 7 Keenan Research Centre, Li Ka Shing Knowledge Institute, St. Michael’s Hospital, 209 Victoria Street, Toronto, ON M5B 1T8 Canada

**Keywords:** Electrical activity of the diaphragm, Respiratory drive, Esophageal pressure, Inspiratory effort, Work of breathing, Respiratory pattern, Assisted ventilation

## Abstract

**Background:**

The electrical activity of the crural diaphragm (Eadi), a surrogate of respiratory drive, can now be measured at the bedside in mechanically ventilated patients with a specific catheter. The expected range of Eadi values under stressed or assisted spontaneous breathing is unknown. This study explored Eadi values in healthy subjects during unstressed (baseline), stressed (with a resistance) and assisted spontaneous breathing. The relation between Eadi and inspiratory effort was analyzed.

**Methods:**

Thirteen healthy male volunteers were included in this randomized crossover study. Eadi and esophageal pressure (Peso) were recorded during unstressed and stressed spontaneous breathing and under assisted ventilation delivered in pressure support (PS) at low and high assist levels and in neurally adjusted ventilatory assist (NAVA). Overall eight different situations were assessed in each participant (randomized order). Peak, mean and integral of Eadi, breathing pattern, esophageal pressure–time product (PTPeso) and work of breathing (WOB) were calculated offline.

**Results:**

Median [interquartile range] peak Eadi at baseline was 17 [13–22] μV and was above 10 μV in 92% of the cases. Eadi_max_ defined as Eadi measured at maximal inspiratory capacity reached 90 [63 to 99] μV. Median peak Eadi/Eadi_max_ ratio was 16.8 [15.6–27.9]%. Compared to baseline, respiratory rate and minute ventilation were decreased during stressed non-assisted breathing, whereas peak Eadi and PTPeso were increased. During unstressed assisted breathing, peak Eadi decreased during high-level PS compared to unstressed non-assisted breathing and to NAVA (*p* = 0.047). During stressed breathing, peak Eadi was lower during all assisted ventilation modalities compared to stressed non-assisted breathing. During assisted ventilation, across the different conditions, peak Eadi changed significantly, whereas PTPeso and WOB/min were not significantly modified. Finally, Eadi signal was still present even when Peso signal was suppressed due to high assist levels.

**Conclusion:**

Eadi analysis provides complementary information compared to respiratory pattern and to Peso monitoring, particularly in the presence of high assist levels.

*Trial registration* The study was registered as NCT01818219 in clinicaltrial.gov. Registered 28 February 2013

**Electronic supplementary material:**

The online version of this article (10.1186/s13613-019-0564-1) contains supplementary material, which is available to authorized users.

## Background

Assessing inspiratory effort and/or respiratory drive at the bedside is of interest as both too low and too high drive and effort have been recognized as risk factors for lung and/or diaphragmatic injury [1, 2]. Inspiratory effort can be quantified by measuring work of breathing (WOB) and esophageal pressure–time product (PTPeso) using an esophageal balloon-equipped specialized nasogastric tube to measure esophageal pressure [3]. Respiratory drive can be assessed at the bedside as a standard procedure in spontaneously breathing patients, including in acutely ill patients, by measuring the electrical activity of the crural diaphragm (Eadi) [4–7]. This can practically be done using a dedicated catheter equipped with electrodes [8] connected to a recording and signal treatment system implemented in a commercially available ventilator [9–13]. Electrical activity generated in the brainstem respiratory centers is transmitted to the diaphragm through the phrenic nerves and is responsible for diaphragmatic muscle cells depolarization. Global diaphragmatic electrical activity is thus a surrogate of respiratory drive. As Eadi is well correlated with global electrical activity of the diaphragm [4, 6, 14, 15], it can be used to assess respiratory drive and/or respiratory motor neuron recruitment [16, 17]. With the commercially available system, Eadi can be used to pilot the ventilator to provide synchronized and proportional ventilatory assist (neurally adjusted ventilatory assist or NAVA) [12, 13] but also as a monitoring tool on its own [10, 11]. Eadi peak values measured during tidal breathing [18–21] and Eadi peak value over maximal Eadi amplitude measured at maximal inspiratory capacity (Eadi_max_) [4] have previously been used to quantify respiratory drive. Very few data about normal Eadi values recorded and processed by the commercial system are, however, available [18, 22].

The main aim of this study was to explore in healthy subjects the range of values and the changes of Eadi observed during unstressed and stressed (in the presence of an added resistance at the mouth) spontaneous non-assisted breathing and during assisted ventilation delivered in pressure support at low and high assist levels or during NAVA. We wanted to describe the range of values observed, the response to stress or to assistance and the possibility to detect and characterize overassist, a condition in which there is a highly reduced patient’s effort to breathe compared to normal [23, 24]. The second aim of the study was to analyze the correlation between Eadi and techniques used to assess patients’ inspiratory effort derived from esophageal pressure monitoring.

## Methods

### Study design

This study was a physiological randomized, crossover interventional study involving male volunteers. It took place in 2013 in the Department of Medical Intensive Care of the University Hospital of Angers, France. This study was approved by the leading hospital ethics committee (Comité de Protection des Personnes Ouest II) under the reference 2012/35. All participants gave informed consent prior to their inclusion in the study. The study was registered as NCT01818219.

### Participants and sample size

Healthy non-obese (body mass index ≤ 30 kg/m^2^) male volunteers between 18 and 35 years old could participate if they had no contraindication for nasogastric tube insertion. Normal respiratory function was confirmed by lung function tests. In line with similar physiological studies [10, 22], it was decided to include 15 volunteers.

### Recordings and protocol description

**Fig. 1:**
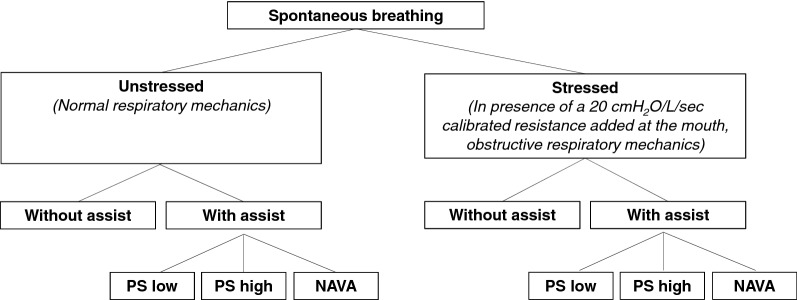
Breathing conditions recorded in a randomized order. *PS* pressure support ventilation, *NAVA* neurally adjusted ventilatory assist
- Recording systems and recorded files
  For all the conditions, Eadi–time curve was recorded using the ventilator-dedicated software (Servo-tracker^®^ software version 4.2, Getinge, Solna, Sweden). Esophageal pressure was recorded using an analog-to-digital converter (MP150, Biopac Systems, Goleta, CA, USA) connected to a pressure transducer and to a laptop computer. Sampling rate for all the recordings was 100 Hz. During assisted ventilation, flow–time curve was recorded using both the ventilator-dedicated software and the analog-to-digital converter (equipped with a flow module connected to a flow sensor placed at the airway opening). The two recording systems were started simultaneously, and data were stored for offline analyses. Synchronization between the two recordings was checked using the time of zero flow at the end of inspiration. Time delay between the corresponding recordings was negligible (< 1 ms). During non-assisted breathing, for technical reasons, the flow–time curve was recorded using the analog-to-digital converter only.

- Preprotocol baseline assessment
  Lung function tests at baseline and in the presence of a calibrated resistance of 20 cmH_2_O/L/s added at the mouth (airway opening) to mimic obstructive respiratory mechanics (stressed breathing) were performed before the beginning of the recordings.
- Placement of the specialized nasogastric tube equipped with electrodes and esophageal balloon and position check
  A nasogastric tube equipped with electrodes and with a 2-mL esophageal balloon placed on the nasogastric tube 13.5 cm proximally from the middle of the electrodes array (Neurovent Research Inc Toronto, Canada) was inserted through the nose to record Eadi–time and esophageal pressure–time curves. Once inserted, the specialized nasogastric tube was connected to a Servo-I^®^ ventilator (Getinge, Solna, Sweden) equipped with a NAVA module (for Eadi recording) and its correct positioning was checked based on Eadi signal with the ventilator built-in module. Consequently to the placement of the electrodes on either side of the diaphragm and because of its position on the nasogastric tube, the esophageal balloon was positioned in the distal third of the esophagus.
- Connection of the recording systems
  The Servo-I^®^ ventilator was connected through a RS 232 cable to a laptop equipped with a dedicated software (Servo-tracker^®^ software version 4.2, Getinge, Solna, Sweden) to record and store Eadi–time, flow–time and pressure–time curves. The esophageal balloon was linked to a pressure transducer for later recordings.
- Subject installation and measurements during a slow inspiration to total lung capacity
  Each volunteer was placed comfortably in a bed in a semi-recumbent position (45°). Flow–time, Eadi–time and esophageal pressure–time curves were recorded during a slow inspiration to total lung capacity [4]. Maximal Eadi value obtained during this maneuver was called Eadi_max_.
- Tested conditions
  Flow–time, Eadi–time and esophageal pressure–time curves were then continuously recorded under eight different breathing conditions (randomized order) (Fig. 1). Recordings were performed under unstressed or stressed spontaneous breathing and without or with assist. Stressed breathing (with an inspiro-expiratory resistance added at the mouth) was used as a model of respiratory disease. Assisted ventilation during unstressed breathing corresponded to a model of overassist. The combination of stressed breathing and assisted ventilation was explored as a third experimental model to assess the combined effect of abnormal respiratory mechanics and assisted ventilation. During spontaneous breathing, the participants were asked to breathe through a mouthpiece wearing a nose clip. Assisted ventilation was delivered through a nasobuccal mask (Vygon, Ecouen, France) tightly strapped on the face to avoid leaks with a Servo-I^®^ ventilator (Getinge, Solna, Sweden). Assist was delivered either using pressure support ventilation (PSV) at low (low PS) or high (high PS) assist levels or using NAVA. For each condition, data were recorded for 10 min during non-assisted breathing (after 15 min of stabilization) and for 5 min during assisted ventilation (after a minimum of 5 min of stabilization without settings modifications). For all the recordings under assisted ventilation, positive end-expiratory pressure (PEEP) was set to 2 cmH_2_O and FIO_2_ to 21%. During PSV, inspiratory trigger was set to 1.2 L/min, inspiratory ramp to 150 ms, and expiratory cycling to 30%. Without the resistance, PSV level was initially set to 2 cmH_2_O during low PS and to 7 cmH_2_O during high PS. In the presence of the resistance, PSV level was initially set to, respectively, 7 and 14 cmH_2_O during low and high PS. For all the PSV steps, in case of intolerance detected during the stabilization phase, PSV level could be reduced. Initial PSV levels were chosen on the basis of what was previously used in healthy volunteers’ studies [22, 25]. During NAVA, the NAVA gain was set to 0.2 cmH_2_O/µV for all the conditions and NAVA trigger was set at 0.5 µV. Backup PSV was activated (settings: flow trigger 1.2 L/min, PSV level 2 cmH_2_O and expiratory cycling 30%).

### Offline analyses

**Fig. 2:**
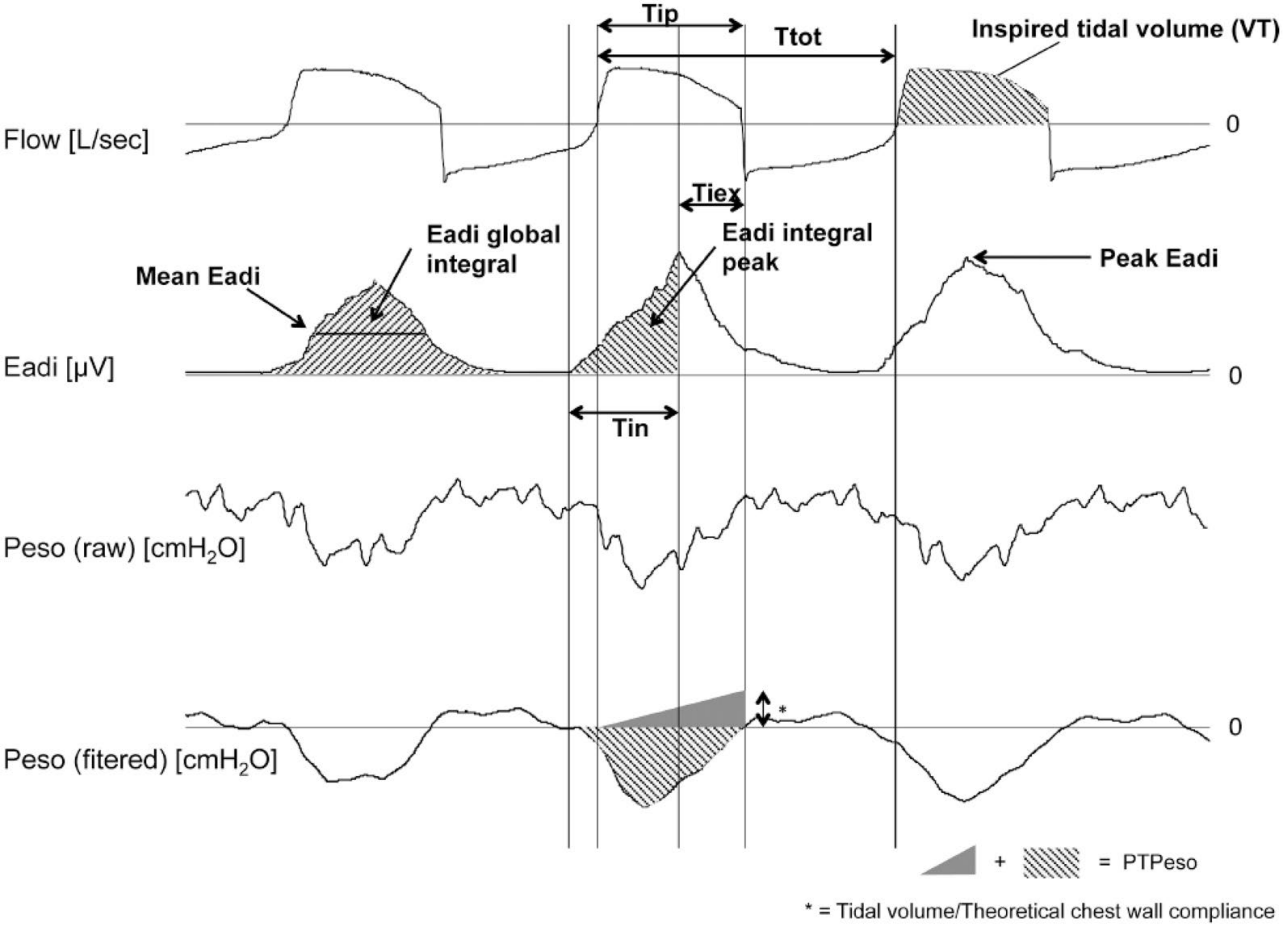
Illustration of the measurements performed offline from the recorded curves (example of assisted ventilation). Tip: Pneumatic inspiratory time, Ttot: Total duration of a respiratory cycle, Tin: neural inspiratory time or time between initial increase in Eadi signal and peak Eadi, Tiex: time delay between maximal Eadi value and end of inspiratory flow. Peak Eadi: maximal Eadi value during one breath, Mean Eadi: mean Eadi value during Eadi increase related to inspiration, Eadi global integral: area under the curve (integral) of the global Eadi signal, Eadi integral peak: area under the curve of Eadi signal censured at the peak, PTPeso: Esophageal pressure–time product

Flow, Eadi and esophageal pressure signal analyses were performed on 25 consecutive breaths after 120 s have elapsed. If an ineffective effort, an esophageal spasm/contraction, an autotriggering or a double triggering [26] was diagnosed by visual inspection, the corresponding respiratory cycle was excluded from the analysis. The Acknowledge software version 4.2 (Biopac Systems, Goleta, CA, USA) was used to analyze Eadi–time and flow–time curves. Measurements derived from esophageal pressure were taken using the SR program (Sistema Respiratorio, a semi-automated research software used in previous works [10, 27]). As illustrated in Fig. 2, for each analyzed breath, inspired tidal volume (VT), pneumatic inspiratory time (Tip)—defined as the duration of inspiratory flow-, and its ratio to total duration of a respiratory cycle (Tip/Ttot) were measured from the flow–time curves. Based on Eadi–time curves, peak, mean Eadi, neural inspiratory time (Tin)—calculated as delta time between initial increase in Eadi and peak Eadi-, integral of the global Eadi signal (Eadi global integral, area under the curve) and integral of Eadi signal censured at the peak (Eadi integral peak) were measured for each analyzed breath (Fig. 2). The ratio of peak Eadi over Eadi_max_ measured at the beginning of the protocol during a slow inspiration to total lung capacity was computed for each analyzed breath (peak Eadi/Eadi_max_). Esophageal pressure time curves were used to measure esophageal pressure time product (PTPeso) and work of breathing (WOB). PTPeso/breath was computed as previously described [28]. PTPeso by minute was computed as the product of mean PTPeso/breath and respiratory rate. WOB was measured using the Campbell diagram [29]. WOB by minute or power was computed as mean WOB/cycle times respiratory rate. WOB indexed for minute ventilation (WOB/L) was computed as mean power divided by minute ventilation in L/min. Overassist was defined as a patient’s effort to breathe highly reduced compared to a normal effort [23, 24], with no visible negative deflection of Peso. Finally, by concomitant analysis of flow–time and Eadi–time curves, the time delay between peak Eadi and the end of inspiratory flow (Tiex) was computed during non-assisted and assisted breathing. By convention, Tiex was considered as positive if peak Eadi occurred earlier than the end of inspiratory flow.

### Statistical methods

The Kolmogorov–Smirnov test was used to assess data distribution. Data were nonparametric and are reported as medians [25th–75th percentile]. Differences between, respectively, two and more than two conditions were assessed using the Wilcoxon test (paired data) or the Friedman analysis and the corresponding post hoc tests for post hoc pairwise comparisons. Of note, when one or more breathing condition(s) could not be recorded in a subject, analysis of variance did not include this subject and the median values mentioned in the text, in the tables and in the figures summarize the values for the participants included in the comparison analysis only. Correlations were assessed using the rank Spearman correlation analysis. Statistical analyses were performed using the MedCalc software version 14.12.0 (Ostend, Belgium). A *p* < 0.05 was considered as statistically significant (bilateral tests).

## Results

Fifteen healthy male volunteers with normal lung function tests were included in the study, but two of them did not tolerate nasogastric tube insertion. All the recordings in spontaneous non-assisted breathing were successfully performed and analyzed. Seventy-one of the 78 (91%) planned recordings under assisted ventilation could be recorded and stored. Due to poor comfort associated with the corresponding conditions, four of the 78 (5.1%) planned sequences under assisted ventilation could not be recorded. Due to automatic switch to the backup PS mode, NAVA could not be applied in one of the participants. For technical reasons, one NAVA recording and two esophageal pressure–time curves (during non-assisted breathing) could not be saved. Finally, in two subjects, the quality of the esophageal pressure curves recorded was not good enough for offline data analyses.

### Subjects’ characteristics, effect of the resistance on the lung function tests, maximal peak Eadi at inspiratory capacity and baseline peak Eadi values

The anthropometric characteristics of the participating subjects are given in Table 1. Lung function tests at baseline were normal. With the resistance, forced expiratory volume during the first second/forced vital capacity (FEV1/FVC) was reduced to 46 [37–55]% of the predicted value. Detailed lung function tests at baseline and with the resistance are provided in Additional file 1: Table S1.

**Table 1:** Subjects’ anthropometric characteristics and recordings performed during a slow inspiration to total lung capacity (TLC)

Subject	Age (years)	Height (m)	Weight (kg)	BMI (kg/m^2^)	Maximal peak Eadi (μV) during slow inspiration to TLC	PTPeso/breath (cmH_2_O*s) during slow inspiration to TLC	Inspiratory capacity (L)
1	22	1.87	105	30.0	47	105.9	2.7
2	30	1.80	70	21.6	123	82.1	2.7
3	30	1.95	82	21.6	104	NA	3.3
4	26	1.83	67	20.0	91	61.1	5.9
5	32	1.71	59	20.2	90	31.6	3.8
6	21	1.78	67	21.1	173	72.2	4.7
7	20	1.67	60	21.5	99	35.8	2.8
8	20	1.71	63	21.5	98	80.5	3.1
9	–	–	–	–	–	–	–
10	30	1.80	65	20.1	46	NA	4.4
11	22	1.80	65	20.1	126	NA	4.3
12	28	1.70	68	23.5	63	72.8	3.9
13	23	1.85	80	23.4	64	95.9	4.2
14	25	1.75	85	27.8	88	92.1	4.0
15	–	–	–	–	–	–	–
Median	25	1.80	67	21.5	91	76.7	3.9
Centile25	22	1.71	65	20.2	64	63.9	3.1
Centile75	30	1.83	80	23.4	104	89.6	4.3

*BMI* body mass index, *Eadi* electrical activity of the diaphragm, *PTPeso* esophageal pressure–time product, *NA* not available

The inspiratory volumes, maximal peak Eadi (Eadi_max_) and PTPeso/breath recorded during a slow inspiration to total lung capacity (maximal inspiration) are given in Table 1. At maximal inspiration, Eadi_max_ reached 91 [64 to 104] μV.

During spontaneous unstressed non-assisted breathing (baseline), peak Eadi values were between 4 and 29 μV. Peak Eadi was above 10 μV in 92% of the recordings and the median peak Eadi was 17 [13–21] μV. Median, minimal and maximal values of peak Eadi/Eadi_max_ ratio are given in Table 2.

**Table 2:** Ratio peak Eadi/Eadi_max_ for the eight different conditions tested

Parameter	Spontaneous breathing (SB)			Assisted breathing without resistance				Assisted breathing with resistance			
	Without resistance	With resistance	p values	PS low	PS high	NAVA	p values	PS low	PS high	NAVA	p values
Peak Eadi/Eadi max (%), median [IQR]	16.8 [15.6–27.9]	24.5 [17.5–35.3]	0.03	12.6 [10.7–33.0]	12.9 [9.3–21.1]	18.3 [14.7–27.3]	*p = 0.047 (*p* < 0.05 between SB and PS high and between NAVA and PS high)	14.9 [13.8–25.6]	22.5 [11.8–30.6]	17.1 [13.6–34.0]	^#^*p* = 0.001 (*p* < 0.05 between SB and PS low, SB and PS high and SB and NAVA)
Minimal peak Eadi/Eadi max (%)	3.5	11.5	–	5.3	1.4	6.7	–	5.5	3.9	2.7	–
Maximal peak Eadi/Eadi max (%)	45.7	47.9	–	45.8	41.5	36.1	–	38.7	52.0	51.3	–

For the multiple comparisons (Friedman analyses), only the subjects for whom all the conditions compared could be recorded were considered. The corresponding median values summarize these patients only

*Eadi* electrical activity of the diaphragm, *Eadi*_*max*_ maximal Eadi amplitude during a voluntary inspiration to total lung capacity, *PS* pressure support, *NAVA* neurally adjusted ventilatory assist

* Comparison between spontaneous non-assisted breathing without resistance and assisted breathing without resistance under PS low, PS high and NAVA

^#^Comparison between spontaneous non-assisted breathing with resistance and assisted breathing with resistance under PS low, PS high and NAVA

### Effect on breathing pattern of adding a resistance or/and assisted ventilation

Adding the resistance during spontaneous non-assisted breathing or applying assisted ventilation without or with the resistance affected minute ventilation (MV) by either modifying respiratory rate (RR), VT or both. Tin and Tiex were also impacted by the different conditions. Oppositely, Ti/Ttot was similar at baseline, in the presence of the resistance and under assisted ventilation without or with the resistance. Median MV, RR, VT, Tin, Tiex and Ti/Ttot are displayed in Table 3 for the eight tested conditions.

**Table 3:** Respiratory profile parameters for the eight different conditions tested

Parameter (median [IQR])	Spontaneous breathing (SB)			Assisted breathing without resistance				Assisted breathing with resistance			
	Without resistance	With resistance	p values	PS low	PS high	NAVA	p values	PS low	PS high	NAVA	p values
MV (L/min)	9.9 (8.7–14.4)	8.9 (7.1–10.5)	*p* = 0.03	13.7 (10.6–18.5)	16.4 (10.7–24.4)	12.9 (11.1–18.5)	*p = 0.001, *p* < 0.05 for all the ventilation modalities compared to SB without resistance *p* > 0.05 for all pairwise comparisons between PS low, PS high and NAVA	11.1 (10.5–12.1)	13.7 (10.9–18.0)	11.1 (9.9–12.5)	^#^*p* = 0.001 *p* < 0.05 for all the ventilation modalities compared to SB with resistance *p* < 0.05 between PS high and PS low and between PS high and NAVA
RR (br/min)	14.6 (12.5–15.7)	10.9 (8.7–12.0)	*p* < 0.001	15.5 (12.9–17.7)	15.3 (13.7–16.5)	14.0 (12.1–17.8)	*p = 0.22	12.7 (10.2–13.6)	12.5 (10.6–13.8)	12.1 (10.4–16.5)	^#^*p* = 0.053
VT (L)	0.81 (0.65–1.00)	0.90 (0.70–1.05)	*p* = 0.54	0.99 (0.79–1.20)	0.99 (0.69–1.71)	0.92 (0.74–1.21)	*p = 0.004 *p* < 0.05 for all the ventilation modalities compared to SB without resistance. *p* > 0.05 for all pairwise comparisons between PS low, PS high and NAVA	0.88 (0.86–1.01)	1.33 (0.96–1.48)	0.90 (0.78–1.04)	^#^*p* = 0.055
Tin (ms)	1.76 (1.48–2.00)	2.41 (1.91–2.67)	*p* < 0.001	1.54 (1.38–1.78)	1.45 (1.36–1.73)	1.68 (1.29–1.91)	*p = 0.46	1.78 (1.63–2.01)	1.78 (1.62–1.96)	1.91 (1.43–2.15)	^#^*p* = 0.027. *p* < 0.05 between SB with resistance and PS low, SB with resistance and PS high and SB with resistance and NAVA
Tiex (ms)	0.17 (0.10–0.22)	0.27 (0.22–0.38)	*p* < 0.001	0.20 (0.14–0.25)	0.24 (0.11–0.29)	0.24 (0.21–0.31)	*p = 0.23	0.31 (0.21–0.33)	0.41 (0.31–0.64)	0.28 (0.20–0.40)	^#^*p* = 0.039. *p* < 0.05 between SB with resistance and PS high, and between PS low and PS high
Ti/Ttot	0.41 (0.40–0.45)	0.44 (0.42–0.47)	*p* = 0.10	0.41 (0.39–0.44)	0.42 (0.37–0.45)	0.44 (0.41–0.47)	*p = 0.17	0.43 (0.40–0.44)	0.41 (0.39–0.45)	0.44 (0.39–0.45)	^#^*p* = 0.97

For the multiple comparisons (Friedman analyses), only the subjects for whom all the conditions compared could be recorded were considered. The corresponding median values summarize these patients only

*MV* minute ventilation, *RR* respiratory rate, *VT* tidal volume, *Tin* neural inspiratory time, *Tiex* inspiratory time in excess, *PS* Pressure support, *NAVA* neurally adjusted ventilatory assist

* Comparison between spontaneous non-assisted breathing without resistance and assisted breathing without resistance under PS low, PS high and NAVA

^#^Comparison between spontaneous non-assisted breathing with resistance and assisted breathing with resistance under PS low, PS high and NAVA

### Effect on Eadi and breathing effort of adding a resistance or/and assisted ventilation

#### Effect of adding the resistance during spontaneous non-assisted breathing

With resistance (stressed breathing), peak Eadi values were between 12 and 39 μV (median value 23 [16–28] μV). Peak Eadi values increased compared to baseline in 10/13 subjects (77%). Overall peak Eadi increased from 16.9 [13.1–21.5] to 22.5 [15.2–28.7] µV (*p* = 0.03) (Additional file 1: Figure S1 A). Median, maximal and minimal peak Eadi/Eadi_max_ ratios are mentioned in Table 2.

PTPeso/min was higher in the presence of the resistance (141.3 [98.4–182.1] compared to 168.7 [128.7–242.1] cmH_2_O × s/min without the resistance, *p* = 0.02) (Additional file 1: Figure S1 B). It increased in 7/10 subjects, but did not apparently increase in three subjects. WOB by minute was not different with and without the resistance (*p* = 0.85), but WOB/L was higher in the presence of the resistance (0.66 [0.48–0.90] versus 0.50 [0.42–0.69] J/L/min, *p* = 0.04).

#### Effect of applying assisted ventilation with PSV (low and high assist levels) and NAVA compared to unstressed non-assisted breathing

**Fig. 3:**
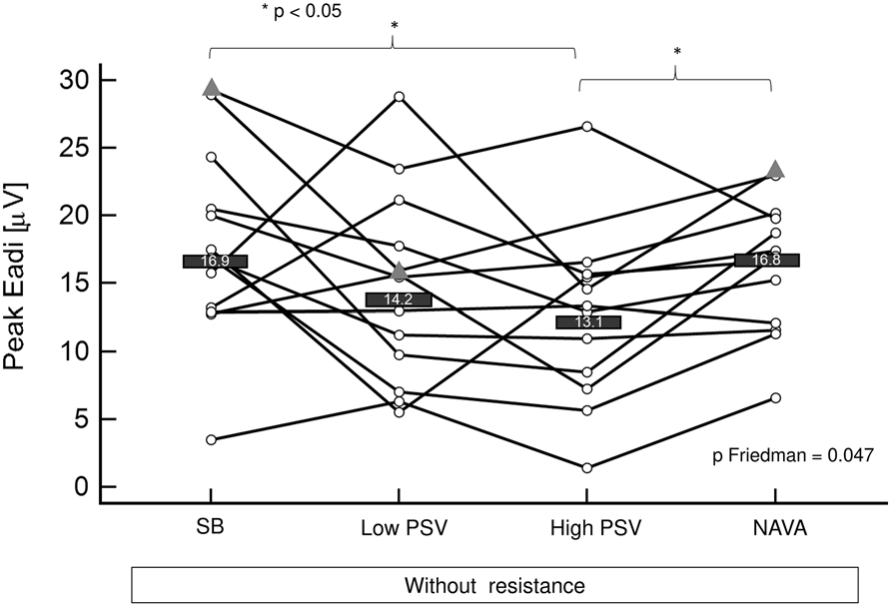
Effect of unstressed assisted ventilation on peak electrical activity of the diaphragm (peak Eadi). *SB* unstressed (without resistance) spontaneous non-assisted breathing, *PSV* pressure support ventilation, *NAVA* neurally adjusted ventilatory assist. The data of all the participants are displayed in the figure. Patients for whom all the conditions were available are represented as white circles. Patients for whom one or more conditions could not be recorded are represented as gray triangles. Only the subjects represented as white circles were considered for median calculations and Friedman analysis

During unstressed assisted breathing, median peak Eadi was lower during high-level PS compared to unstressed non-assisted breathing and to NAVA (*p* = 0.047, *p* < 0.05 for both pairwise comparisons). Of note, when the analysis was performed only for the patients who also had a legible recording of PTPeso, the difference remained significant. There were different effects of the different assisted ventilation modalities across subjects on peak Eadi (Fig. 3). In comparison with low-level PS, peak Eadi decreased in eight subjects during high-level PS. Conversely, it increased in four subjects. In the remaining subject, the high PS level condition could not be recorded because of discomfort. Median, maximal and minimal peak Eadi/Eadi_max_ ratios are mentioned in Table 2.

Frank overassist, indicated by the absence of detectable negative esophageal pressure drop, was noted in 5/10 (50%) of the recordings with high-level PS. In these recordings, positive Eadi deflections were low but still visible. A representative recording illustrating this situation is displayed in Additional file 1: Figure S2. This was not observed with low-level PS or NAVA.

**Fig. 4:**
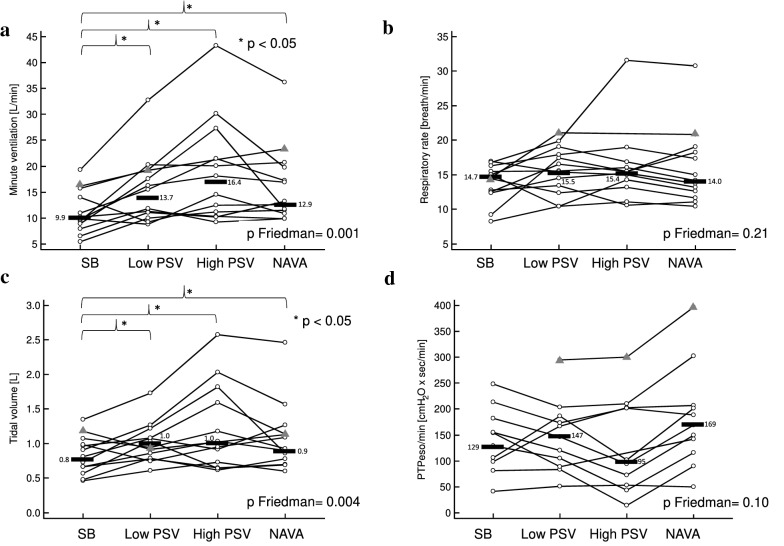
Effect of unstressed assisted ventilation on MV (**a**), RR (**b**), VT (**c**) and PTPeso/min (**d**). *MV* minute ventilation, *RR* respiratory rate, *VT* tidal volume, *PTPeso/min* esophageal pressure–time product by minute, *SB* unstressed (without resistance) spontaneous non-assisted breathing, *PSV* pressure support ventilation, *NAVA* neurally adjusted ventilator assist. The data of all the participants are displayed in the figure. Patients for whom all the conditions were available are represented as white circles. Patients for whom one or more conditions could not be recorded are represented as gray triangles. Only the patients represented as white circles were considered for median calculations and Friedman analyses

By contrast with Eadi, PTPeso/min (*p* Friedman 0.10) and WOB/min (*p* Friedman 0.14) were not different during unstressed non-assisted breathing and under the different modalities of assisted ventilation without resistance (see individual data for PTPeso/min, Fig. 4d). WOB/L was slightly lower during high-level PS compared to NAVA but not different between high-level PS and low-level PS or unstressed non-assisted breathing. Median values were 0.34 [0.16–0.66] J/L during high-level PS, 0.63 [0.40–0.71] J/L during NAVA, 0.54 [0.38–0.63] J/L during low-level PS and 0.44 [0.31–0.69] J/L during unstressed non-assisted breathing. (*p* Friedman = 0.04 with significant pairwise comparison between NAVA and high-level PS only.)

#### Effect of applying assisted ventilation with PSV (low and high assist levels) and NAVA in the presence of the resistance compared to stressed non-assisted breathing

**Fig. 5:**
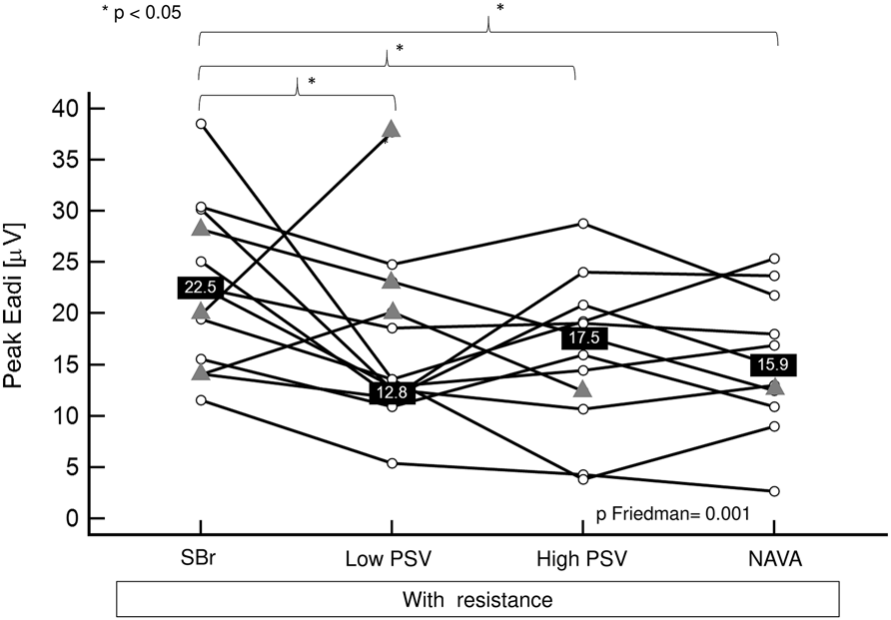
Effect of stressed assisted ventilation on peak electrical activity of the diaphragm (peak Eadi). *SBr* stressed (with resistance) spontaneous non-assisted breathing, *PSV* pressure support ventilation, *NAVA* neurally adjusted ventilatory assist. The data of all the participants are displayed in the figure. Patients for whom all the conditions were available are represented as white circles. Patients for whom one or more conditions could not be recorded are represented as gray triangles. Only the patients represented as white circles were considered for median calculations and Friedman analysis

Overall, during resistive breathing, peak Eadi was significantly decreased during all the modalities of assisted ventilation compared to stressed non-assisted breathing (*p* < 0.001, with significant pairwise comparisons for all modes). Of note, when the analysis was performed only for the patients who also had a legible recording of PTPeso, the difference remained significant (*p* < 0.001). In the presence of the resistance, the effect of applying the different modalities of assisted ventilation on peak Eadi varied across subjects (Fig. 5). Peak Eadi was lower in all subjects during high-level PS ventilation compared to stressed non-assisted breathing. In comparison with low-level PS, peak Eadi decreased in four subjects during high-level PS. Conversely, it increased in seven subjects. In the two remaining subjects, the high PS level condition could not be recorded because of discomfort. Median, maximal and minimal peak Eadi/Eadi_max_ ratios are mentioned in Table 2.

During resistive breathing, frank overassist was noted in 5/9 (55.6%) recordings with high-level PS, in 1/10 (10%) recordings with low-level PS and never with NAVA. Eadi was still visible in all cases.

**Fig. 6:**
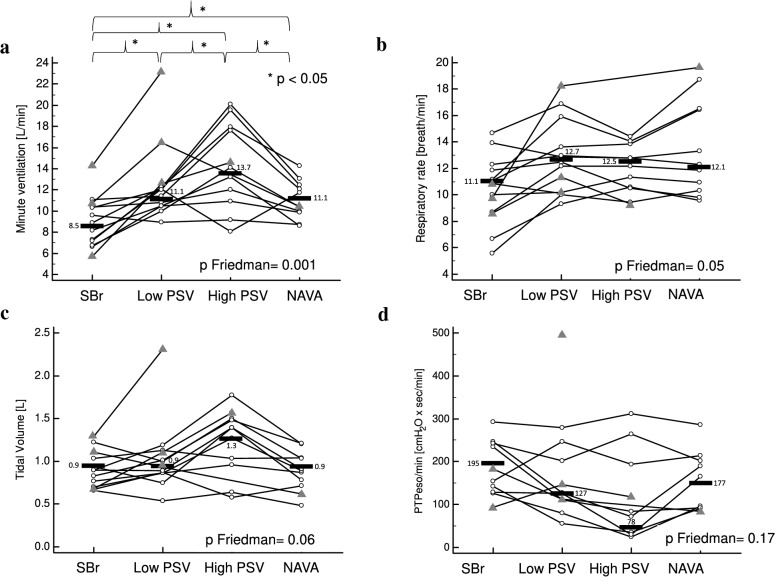
Effect of stressed assisted ventilation on MV (**a**), RR (**b**), VT (**c**) and PTPeso/min (**d**). *MV* minute ventilation, *RR* respiratory rate, *VT* tidal volume, *PTPeso/min* esophageal pressure–time product by minute. *SBr* spontaneous non-assisted breathing in the presence of the resistance, *PSV* pressure support ventilation. *NAVA* neurally adjusted ventilatory assist. The data of all the participants are displayed in the figure. Patients for whom all the conditions were available are represented as white circles. Patients for whom one or more conditions could not be recorded are represented as gray triangles. Only the patients represented as white circles were considered for median calculations and Friedman analyses

During resistive breathing, PTPeso/min (Fig. 6d), WOB/min and WOB/L were not significantly different during the various modalities of assisted ventilation and during stressed non-assisted breathing (*p* Friedman 0.17, 0.30 and 0.11, respectively).

### Correlations between indexes

For all the recorded conditions and for all the subjects considered together (breath-by-breath correlation), the Spearman rho coefficients of correlations between peak Eadi, Eadi global integral, Eadi integral peak and mean Eadi were between 0.86 and 0.94 (*p* < 0.001). Breath-by-breath correlation between peak Eadi and PTPeso/cycle showed a rho coefficient at 0.43 (*p* < 0.001, *N* = 1639 breathes). When correlation between peak Eadi and PTPeso/cycle was assessed independently for each subject, the rho coefficients were between 0.41 and 0.83 with a median value of 0.57 [0.53–0.71]. The rho coefficient of the correlation between peak Eadi mean value and PTPeso/min was 0.43 (*p* < 0.001, *n* = 81).

## Discussion

Our study demonstrated that median peak Eadi normal baseline values are around 15 μV and in most cases above 10 μV but within a broad range. Peak Eadi, the easiest Eadi parameter to monitor at the bedside, was well correlated with Eadi integral and mean Eadi. During stressed (with resistance) non-assisted breathing, peak Eadi increased compared to unstressed non-assisted breathing. Overall, assisted ventilation induced a decrease in peak Eadi compared to non-assisted breathing both during unstressed (without resistance) and stressed breathing. In the same conditions, changes in breathing effort assessed by Peso were often nonsignificant. In some conditions there was a paradoxical increase in peak Eadi when the level of assist was increased, underlining that Eadi could provide information not available using standard respiratory monitoring or esophageal pressure monitoring. We finally demonstrated that Eadi signal was still present when, in the presence of high assist levels, no esophageal deflections were visible on the esophageal pressure–time curve.

Previous data on normal baseline (i.e., during spontaneous unstressed non-assisted breathing) peak Eadi are very sparse [18, 22], but in line with ours recorded in healthy volunteers. The wide range of peak Eadi and of peak Eadi/Eadi_max_ ratios values at baseline could be explained by differences in the subjects’ anatomical characteristics as the distance between the nasogastric tube electrodes and the crural diaphragm was previously reported as having an influence on absolute Eadi values [14, 30]. In addition, it is important to keep in mind that in patients other factors as, for example, the subject’s age or the presence of an underlying chronic lung condition could also affect peak Eadi amplitude [18] meaning that, for a given neural output, peak Eadi amplitude could differ. It must also be said that Eadi is a processed signal [4, 31, 32]. As described by Sinderby et al., normalizing Eadi amplitude to the maximal amplitude obtained during a volunteer inspiration from functional residual capacity to total lung capacity could help defining Eadi thresholds usable for monitoring [4]. Acutely ill patients, however, are often not able to perform a maximal inspiration on request. Electromagnetic stimulation of the phrenic nerve [18] is a reliable alternative method to normalize Eadi amplitude in critically ill patients but is only available for research purposes. Interestingly, our results showed that peak Eadi and peak Eadi/Eadi_max_ ratio gave the same information.

During stressed (with resistance) spontaneous breathing, MV decreased compared to baseline (i.e., spontaneous non-assisted breathing without resistance) due to RR decrease. Tin increased, while VT remained stable. This is in line with previous data reported by Calabrese et al. [33] except for VT, which did not significantly increase in our study in the presence of the resistance. Peak Eadi values (23 [16–28] μV) were higher compared to baseline in the presence of the resistance. They can be compared to peak Eadi recorded during non-assisted breathing in patients suffering from various respiratory disease. Jolley et al. [18] reported mean peak Eadi values in stable not ventilated COPD patients at 53 ± 29 μV. Peak Eadi values reported in ventilated patients recovering from respiratory failure during a spontaneous breathing trial [19, 20, 34] were in general close to the values recorded in our model. Ferreira et al. [21] reported, however, lower peak Eadi of 10 [6–21] μV during a 5-cmH_2_O pressure support weaning test. Our study showed an absence of concordance between VT and Eadi variations when unstressed and stressed non-assisted spontaneous breathings were compared. Our study clearly demonstrates that respiratory drive per breath may increase even if RR decreases, which cannot be diagnosed using standard respiratory monitoring. As expected, an increase in PTPeso/min and WOB/L occurred when inspiratory load increased. An increase in peak Eadi was overall correlated with the increase in PTPeso/min and WOB/L. This is in line with the data published by Bellani et al. [9] who demonstrated a good correlation between muscular pressure and Eadi amplitude. Interestingly, the breath-by-breath correlation between Eadi amplitude and PTPeso was relatively poor in our study, suggesting that Eadi provides different information for a given breath compared to PTPeso. This information is new as only mean or median values of Eadi- and Peso-derived monitoring parameters were compared in previous works [9, 35–37].

Eadi amplitude decreased when assisted breathing was compared to unstressed non-assisted breathing. In this situation, assisted ventilation induced an increase in MV due to VT increase without concomitant RR increase, in line with the data of Meric et al. [22] recorded in healthy volunteers receiving PSV. In accordance with what was previously described in ventilated patients suffering from acute respiratory failure [20, 38, 39], median peak Eadi also decreased during resistive breathing (stressed breathing) when assisted ventilation was applied. In our volunteers, however, both in the presence and in the absence of the resistance, the effect on Eadi amplitude of applying the different modalities of assisted ventilation varied across subjects and decrease in Eadi amplitude was not always observed when assist level was increased. Interestingly, we even observed in some of our subjects a paradoxical increase in peak Eadi when high-level PS was applied. This has been shown before in healthy subjects receiving noninvasive ventilation through an helmet [40] and could potentially be due to overassist-related discomfort associated with increase in respiratory drive. This observation again suggests that Eadi monitoring can provide information that cannot be obtained by standard respiratory or by esophageal pressure monitoring. It must, however, be underlined that paradoxical increase in peak Eadi under high-level PS could also be explained by another phenomenon. Indeed, Eadi amplitude can, at least for supramaximal phrenic nerve stimulation, be influenced by changes in lung volumes [41]. Overdistention-related diaphragm flattening could thus contribute to explain paradoxical increase in peak Eadi during high-level PS (changes in the distance between the array of electrodes and the diaphragm pillars). This hypothesis has, however, as far as we know, not been tested for lung volumes close to tidal volumes.

Eadi signal was still present even when the negative pressure drops were no longer visible on the esophageal pressure–time curve due to very low effort in presence of high assist level. The interest of Eadi monitoring in diagnosing overassist was already suggested by Sinderby et al. [36] and Carteaux et al. [20] who reported examples of overassist with no detectable PTPeso drop but a persistent Eadi signal, respectively, in healthy volunteers and in patients recovering from respiratory failure. Our data confirm that Eadi monitoring could help to distinguish overassist from passive insufflation or autotriggering. As overassist is a frequent [23], difficult to diagnose and potentially deleterious condition during assisted ventilation [1, 24]), detecting overassist could be one of the clinical interests of extending the use of Eadi monitoring. In addition, as Eadi could always be recorded in ventilated patients, our data might suggest that Eadi monitoring could be interesting in case of severe respiratory muscle weakness or any case of dissociation between drive and effort.

### Limitations

Several limitations must be mentioned. First, the respiratory profile at baseline (spontaneous non-assisted breathing without resistance) could have been influenced by the experimental setting [42] and the use of a mouth piece with a small intrinsic resistance to record inspiratory and expiratory flow. For example, some of the subjects had relatively high tidal volumes, suggesting that the baseline respiratory pattern potentially differed from a standard respiratory profile at rest. Second, the nasogastric tube was positioned in order to have the Eadi electrodes well positioned using a previously described technique [43]. Consequently to the placement of the electrodes on either side of the diaphragm and because of its position on the nasogastric tube, the esophageal balloon was positioned in the distal third of the esophagus. The correct placement of the esophageal balloon was not systematically checked with an occlusion maneuver. However, an occlusion maneuver was performed in three of the participants and showed a very good concordance between airway pressure and esophageal pressure variations during an inspiratory effort against occlusion, thus confirming the correct placement of the balloon in these three subjects. Third, the levels of assist used during assisted ventilation were chosen arbitrarily. Fourth, in the presence of the resistance, PTPeso/min increased in only 7/10 subjects, which could suggest that the resistance used was not big enough to create significant stress in all the subjects. It cannot be excluded that different results could have been found under different experimental conditions. Fifth, the present study was performed in healthy, awake subjects who could have a regulation of breathing different from sedated or sleeping patients. In other words, this study provides ranges of Eadi values in healthy volunteers. It cannot be excluded that values recorded in ventilated patients differ due for example to sedation analgesia treatment or to abnormally increased respiratory drive in response to impaired respiratory muscle strength. Overall, this study was performed in healthy volunteers under very specific experimental conditions. Extrapolation of our results to ICU patients remains to be demonstrated. When drawing clinical conclusion from our results, caution is thus warranted.

## Conclusions

Our study demonstrated that peak Eadi normal values were within a broad range but usually above 10 µV. Our model illustrates examples of situations where Eadi amplitude does not change in parallel to PTPeso suggesting that Eadi monitoring provides additional and different information compared to respiratory pattern and to esophageal pressure monitoring. Using Eadi as advanced respiratory monitoring could thus be of interest to better integrate breathing regulation in our clinical assessment.

## Additional file

**Additional file 1: Table S1.** Detailed lung function tests at baseline and in the presence of the 20 cmH2O/L/sec resistance. FVC: Forced Vital capacity, FEV1: Forced expiratory volume in one second. PEF: Peak expiratory flow. Res: Resistance. **Figure S1.** Effect of a resistance on peak Eadi (A) and PTPeso/min (B) during spontaneous non-assisted breathing. PTPeso/minute: esophageal pressure-time product by minute. Eadi: Electrical activity of the diaphragm. The middle line of box-and-whisker plot represents the median. The central box represents the values from the lower to upper quartile (25 to 75 percentile). The vertical line extends from the minimum to the maximum values. **Figure S2.** Over-assist during pressure support ventilation (PSV) indicated by absence of visible negative esophageal pressure drop. Note that peak electrical activity of the diaphragm (peak Eadi) is on average below 6 microvolts. Eso: esophageal.

## Data Availability

The datasets used and/or analyzed during the current study are available from the corresponding author on reasonable request.

## Abbreviations

Eadi: electrical activity of the diaphragm
Eadi_max_,: maximal Eadi value measured during a slow inspiration to total lung capacity
Eadi global integral: integral of the global Eadi signal
Eadi integral peak: Eadi signal integral censured at the peak
FEV1: forced expiratory volume during the first second
FVC: forced vital capacity
High PS: high level of pressure support
Low PS: low level of pressure support
MV: minute ventilation
NAVA: neutrally adjusted ventilatory assist
PEEP: positive end-expiratory pressure
Peso: esophageal pressure
PTPeso: esophageal pressure–time product
PS: pressure support
PSV: pressure support ventilation
RR: respiratory rate
Tiex: time delay between peak Eadi and the end of inspiratory flow
Tin: neural inspiratory time
Tip: pneumatic inspiratory time
Ttot: total duration of a respiratory cycle
VT: inspired tidal volume
WOB: work of breathing

## References

[1] Goligher EC, Dres M, Fan E, Rubenfeld GD, Scales DC, Herridge MS, Vorona S, Sklar MC, Rittayamai N, Lanys A, Murray A, Brace D, Urrea C, Reid WD, Tomlinson G, Slutsky AS, Kavanagh BP, Brochard LJ, Ferguson ND (2018). Mechanical ventilation-induced diaphragm atrophy strongly impacts clinical outcomes. Am J Respir Crit Care Med.

[2] Brochard L, Slutsky A, Pesenti A (2017). Mechanical ventilation to minimize progression of lung injury in acute respiratory failure. Am J Respir Crit Care Med.

[3] Akoumianaki E, Maggiore SM, Valenza F, Bellani G, Jubran A, Loring SH, Pelosi P, Talmor D, Grasso S, Chiumello D, Guerin C, Patroniti N, Ranieri VM, Gattinoni L, Nava S, Terragni PP, Pesenti A, Tobin M, Mancebo J, Brochard L, Group PW (2014). The application of esophageal pressure measurement in patients with respiratory failure. Am J Respir Crit Care Med.

[4] Sinderby C, Beck J, Spahija J, Weinberg J, Grassino A (1998). Voluntary activation of the human diaphragm in health and disease. J Appl Physiol.

[5] Lopata M, Evanich MJ, Lourenco RV (1977). Relationship between mouth occlusion pressure and electrical activity of the diaphragm: effects of flow-resistive loading. Am Rev Respir Dis.

[6] Oyer LM, Knuth SL, Ward DK, Bartlett D (1989). Patterns of neural and muscular electrical activity in costal and crural portions of the diaphragm. J Appl Physiol.

[7] Petit JM, Milic-Emili G (1959). Delhez L [New technic for the study of functions of the diaphragmatic muscle by means of electromyography in man]. Boll Soc Ital Biol Sper.

[8] Sinderby C, Navalesi P, Beck J, Skrobik Y, Comtois N, Friberg S, Gottfried SB, Lindstrom L (1999). Neural control of mechanical ventilation in respiratory failure. Nat Med.

[9] Bellani G, Mauri T, Coppadoro A, Grasselli G, Patroniti N, Spadaro S, Sala V, Foti G, Pesenti A (2013). Estimation of patient’s inspiratory effort from the electrical activity of the diaphragm. Crit Care Med.

[10] Beloncle F, Piquilloud L, Rittayamai N, Sinderby C, Roze H, Brochard L (2017). A diaphragmatic electrical activity-based optimization strategy during pressure support ventilation improves synchronization but does not impact work of breathing. Crit Care.

[11] Colombo D, Cammarota G, Alemani M, Carenzo L, Barra FL, Vaschetto R, Slutsky AS, Della Corte F, Navalesi P (2011). Efficacy of ventilator waveforms observation in detecting patient-ventilator asynchrony. Crit Care Med.

[12] Piquilloud L, Tassaux D, Bialais E, Lambermont B, Sottiaux T, Roeseler J, Laterre PF, Jolliet P, Revelly JP (2012). Neurally adjusted ventilatory assist (NAVA) improves patient-ventilator interaction during non-invasive ventilation delivered by face mask. Intensive Care Med.

[13] Piquilloud L, Vignaux L, Bialais E, Roeseler J, Sottiaux T, Laterre PF, Jolliet P, Tassaux D (2011). Neurally adjusted ventilatory assist improves patient-ventilator interaction. Intensive Care Med.

[14] Beck J, Sinderby C, Lindstrom L, Grassino A (1985). Effects of lung volume on diaphragm EMG signal strength during voluntary contractions. J Appl Physiol.

[15] Nava S, Ambrosino N, Crotti P, Fracchia C, Rampulla C (1993). Recruitment of some respiratory muscles during three maximal inspiratory manoeuvres. Thorax.

[16] Lourenco RV, Cherniack NS, Malm JR, Fishman AP (1966). Nervous output from the respiratory center during obstructed breathing. J Appl Physiol.

[17] Belman MJ, Sieck GC (1982). The ventilatory muscles. Fatigue, endurance and training. Chest.

[18] Jolley CJ, Luo YM, Steier J, Reilly C, Seymour J, Lunt A, Ward K, Rafferty GF, Polkey MI, Moxham J (2009). Neural respiratory drive in healthy subjects and in COPD. Eur Respir J.

[19] Roze H, Lafrikh A, Perrier V, Germain A, Dewitte A, Gomez F, Janvier G, Ouattara A (2011). Daily titration of neurally adjusted ventilatory assist using the diaphragm electrical activity. Intensive Care Med.

[20] Carteaux G, Cordoba-Izquierdo A, Lyazidi A, Heunks L, Thille AW, Brochard L (2016). Comparison between neurally adjusted ventilatory assist and pressure support ventilation levels in terms of respiratory effort. Crit Care Med.

[21] Ferreira JC, Diniz-Silva F, Moriya HT, Alencar AM, Amato MBP, Carvalho CRR (2017). Neurally adjusted ventilatory assist (NAVA) or pressure support ventilation (PSV) during spontaneous breathing trials in critically ill patients: a crossover trial. BMC Pulm Med..

[22] Meric H, Calabrese P, Pradon D, Lejaille M, Lofaso F, Terzi N (2014). Physiological comparison of breathing patterns with neurally adjusted ventilatory assist (NAVA) and pressure-support ventilation to improve NAVA settings. Respir Physiol Neurobiol.

[23] Pletsch-Assuncao R, Caleffi Pereira M, Ferreira JG, Cardenas LZ, de Albuquerque ALP, de Carvalho CRR, Caruso P (2018). Accuracy of invasive and noninvasive parameters for diagnosing ventilatory overassistance during pressure support ventilation. Crit Care Med.

[24] Brochard L, Telias I (2018). Bedside detection of overassistance during pressure support ventilation. Crit Care Med.

[25] Scheid P, Lofaso F, Isabey D, Harf A (1985). Respiratory response to inhaled CO_2_ during positive inspiratory pressure in humans. J Appl Physiol.

[26] Thille AW, Rodriguez P, Cabello B, Lellouche F, Brochard L (2006). Patient-ventilator asynchrony during assisted mechanical ventilation. Intensive Care Med.

[27] L’Her E, Deye N, Lellouche F, Taille S, Demoule A, Fraticelli A, Mancebo J, Brochard L (2005). Physiologic effects of noninvasive ventilation during acute lung injury. Am J Respir Crit Care Med.

[28] Aslanian P, El Atrous S, Isabey D, Valente E, Corsi D, Harf A, Lemaire F, Brochard L (1998). Effects of flow triggering on breathing effort during partial ventilatory support. Am J Respir Crit Care Med.

[29] Cabello B, Mancebo J (2006). Work of breathing. Intensive Care Med.

[30] Beck J, Sinderby C, Weinberg J, Grassino A (1995). Effects of muscle-to-electrode distance on the human diaphragm electromyogram. J Appl Physiol.

[31] Sinderby CA, Beck JC, Lindstrom LH, Grassino AE (1985). Enhancement of signal quality in esophageal recordings of diaphragm EMG. J Appl Physiol.

[32] American Thoracic Society/European Respiratory S (2002). ATS/ERS Statement on respiratory muscle testing. Am J Respir Crit Care Med.

[33] Calabrese P, Dinh TP, Eberhard A, Bachy JP, Benchetrit G (1998). Effects of resistive loading on the pattern of breathing. Respir Physiol.

[34] Muttini S, Villani PG, Trimarco R, Bellani G, Grasselli G, Patroniti N (2015). Relation between peak and integral of the diaphragm electromyographic activity at different levels of support during weaning from mechanical ventilation: a physiologic study. J Crit Care.

[35] Brander L, Leong-Poi H, Beck J, Brunet F, Hutchison SJ, Slutsky AS, Sinderby C (2009). Titration and implementation of neurally adjusted ventilatory assist in critically ill patients. Chest.

[36] Sinderby C, Beck J, Spahija J, de Marchie M, Lacroix J, Navalesi P, Slutsky AS (2007). Inspiratory muscle unloading by neurally adjusted ventilatory assist during maximal inspiratory efforts in healthy subjects. Chest.

[37] Mauri T, Grasselli G, Suriano G, Eronia N, Spadaro S, Turrini C, Patroniti N, Bellani G, Pesenti A (2016). Control of respiratory drive and effort in extracorporeal membrane oxygenation patients recovering from severe acute respiratory distress syndrome. Anesthesiology.

[38] Beck J, Gottfried SB, Navalesi P, Skrobik Y, Comtois N, Rossini M, Sinderby C (2001). Electrical activity of the diaphragm during pressure support ventilation in acute respiratory failure. Am J Respir Crit Care Med.

[39] Patroniti N, Bellani G, Saccavino E, Zanella A, Grasselli G, Isgro S, Milan M, Foti G, Pesenti A (2012). Respiratory pattern during neurally adjusted ventilatory assist in acute respiratory failure patients. Intensive Care Med.

[40] Moerer O, Beck J, Brander L, Costa R, Quintel M, Slutsky AS, Brunet F, Sinderby C (2008). Subject-ventilator synchrony during neural versus pneumatically triggered non-invasive helmet ventilation. Intensive Care Med.

[41] Gandevia SC, McKenzie DK (1985). Human diaphragmatic EMG: changes with lung volume and posture during supramaximal phrenic stimulation. J Appl Physiol.

[42] Gilbert R, Auchincloss JH, Brodsky J, Boden W (1972). Changes in tidal volume, frequency, and ventilation induced by their measurement. J Appl Physiol.

[43] Barwing J, Ambold M, Linden N, Quintel M, Moerer O (2009). Evaluation of the catheter positioning for neurally adjusted ventilatory assist. Intensive Care Med.

