# Supplementary material for: Information conveyed by electrical diaphragmatic activity during unstressed, stressed and assisted spontaneous breathing: a physiological study
Source: Ann Intensive Care. 2019 Aug 14;9:89. doi: 10.1186/s13613-019-0564-1 (PMC6692797; doi:10.1186/s13613-019-0564-1)
Supplement: Supplementary file 1 — Additional file 1: Table S1. Detailed lung function tests at baseline and in the presence of the 20 cmH2O/L/sec resistance. FVC: Forced Vital capacity, FEV1: Forced expiratory volume in one second. PEF: Peak expiratory flow. Res: Resistance. Figure S1. Effect of a resistance on peak Eadi (A) and PTPeso/min (B) during spontaneous non-assisted breathing. PTPeso/minute: esophageal pressure-time product by minute. Eadi: Electrical activity of the diaphragm. The middle line of box-and-whisker plot represents the median. The central box represents the values from the lower to upper quartile (25 to 75 percentile). The vertical line extends from the minimum to the maximum values. Figure S2. Over-assist during pressure support ventilation (PSV) indicated by absence of visible negative esophageal pressure drop. Note that peak electrical activity of the diaphragm (peak Eadi) is on average below 6 microvolts. Eso: esophageal. [file 13613_2019_564_MOESM1_ESM.docx]

**Information conveyed by electrical diaphragmatic activity during unstressed, stressed and assisted spontaneous breathing: a physiologic study.**

**Additional File**

**Results, additional data**

Detailed lung function tests at baseline and in the presence of a 20 cmH_2_O/L/sec inspiro-expiratory resistance are provided in table E1.

| **Subject** | **FVC [L]** | | **FVC [%predicted]** | | **FEV1/FVC [%predicted]** | | **FEV1 [L]** | | **FEV1 [%predicted]** | | **PEF [L/s]** | | **PEF [%predicted]** | |
| --- | --- | --- | --- | --- | --- | --- | --- | --- | --- | --- | --- | --- | --- | --- |
|  | *Baseline* | *With Res.* | *Baseline* | *With Res.* | *Baseline* | *With Res.* | *Baseline* | *With Res.* | *Baseline* | *With Res.* | *Baseline* | *With Res.* | *Baseline* | *With Res.* |
| **1** | 6.1 | 5.1 | 102 | 86 | 94 | 36 | 4.7 | 1.5 | 100 | 32 | 9.5 | 1.6 | 91 | 15 |
| **2** | 6.2 | 5.9 | 112 | 106 | 96 | 45 | 4.8 | 2.1 | 110 | 49 | 10.3 | 2.3 | 104 | 23 |
| **3** | 6.2 | 6.3 | 98 | 99 | 91 | 42 | 4.6 | 2.1 | 92 | 43 | 9.4 | 2.2 | 87 | 21 |
| **4** | 5 | 5 | 87 | 88 | 106 | 31 | 4.4 | 1.3 | 96 | 28 | 8.7 | 1.3 | 85 | 13 |
| **5** | 4.9 | 3.9 | 101 | 81 | 95 | 37 | 3.7 | 1.2 | 93 | 30 | 8.4 | 0.9 | 91 | 10 |
| **6** | 5.6 | 5.9 | 102 | 108 | 94 | 46 | 4.4 | 2.2 | 99 | 50 | 8.9 | 2.4 | 89 | 23 |
| **7** | 4.3 | 4.2 | 90 | 88 | 97 | 56 | 3.5 | 2 | 87 | 49 | 8.7 | 2.4 | 93 | 25 |
| **8** | 4.2 | 4.9 | 81 | 97 | 92 | 55 | 3.1 | 2.3 | 75 | 54 | 7 | 2.6 | 72 | 27 |
| **9** | - | - | - | - | - | - | - | - | - | - | - | - | - | - |
| **10** | 5.8 | 5.2 | 106 | 95 | 82 | 69 | 4.5 | 2.5 | 103 | 57 | 8.7 | 2.5 | 88 | 26 |
| **11** | 5.2 | 5.2 | 92 | 93 | 113 | 60 | 4.8 | 2.6 | 106 | 58 | 5.2 | 2.7 | 92 | 27 |
| **12** | 4.6 | 5 | 93 | 100 | 90 | 48 | 3.4 | 1.9 | 85 | 48 | 7.6 | 2.2 | 81 | 23 |
| **13** | 5.2 | - | 87 | - | 93 | - | 4 | - | 84 | - | 8 | - | 76 | - |
| **14** | 5.6 | 5.4 | 105 | 101 | 83 | 22 | 3.9 | 1.9 | 91 | 22 | 8.6 | 1.8 | 87 | 18 |
| **15** | - | - | - | - | - | - | - | - | - | - | - | - | - | - |
| **Median** | **5.2** | **5.2** | **98** | **96** | **94** | **46** | **4.4** | **2.1** | **93** | **49** | **8.7** | **2.3** | **88** | **23** |
| **Centile 25** | **4.9** | **5** | **90** | **88** | **91** | **37** | **3.7** | **1.8** | **87** | **32** | **8** | **1.8** | **85** | **17** |
| **Centile 75** | **5.8** | **5.5** | **102** | **100** | **96** | **55** | **4.6** | **2.2** | **100** | **51** | **8.9** | **2.4** | **91** | **25** |

**Table S1.** Detailed lung function tests at baseline and in the presence of a 20 cmH_2_O/L/sec inspiro-expiratory resistance. FVC: Forced Vital capacity, FEV1: Forced expiratory volume in one second. PEF: Peak expiratory flow. Res: Resistance

Graphical illustration of the effect of adding a 20 cmH_2_O/L/sec inspiro-expiratory resistance on minute ventilation, respiratory rate, peak Eadi and PTPeso/minute.


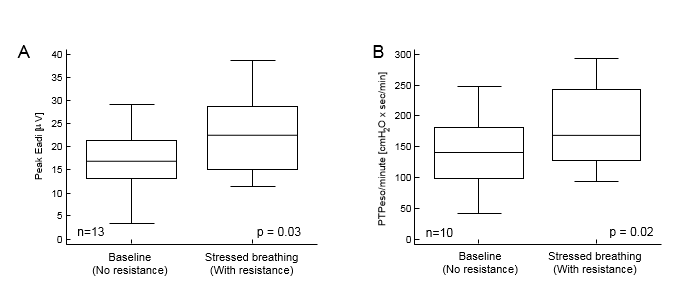


**Figure S1.** Effect of a resistance on peak Eadi (A) and PTPeso/min (B) during spontaneous non-assisted breathing. PTPeso/min: esophageal pressure-time product by minute. Eadi: Electrical activity of the diaphragm. The middle line of box-and-whisker plot represents the median. The central box represents the values from the lower to upper quartile (25 to 75 percentile). The vertical line extends from the minimum to the maximum values.

Illustration of frank over-assist during assisted ventilation indicated by the absence of negative esophageal pressure drop during assisted ventilation.


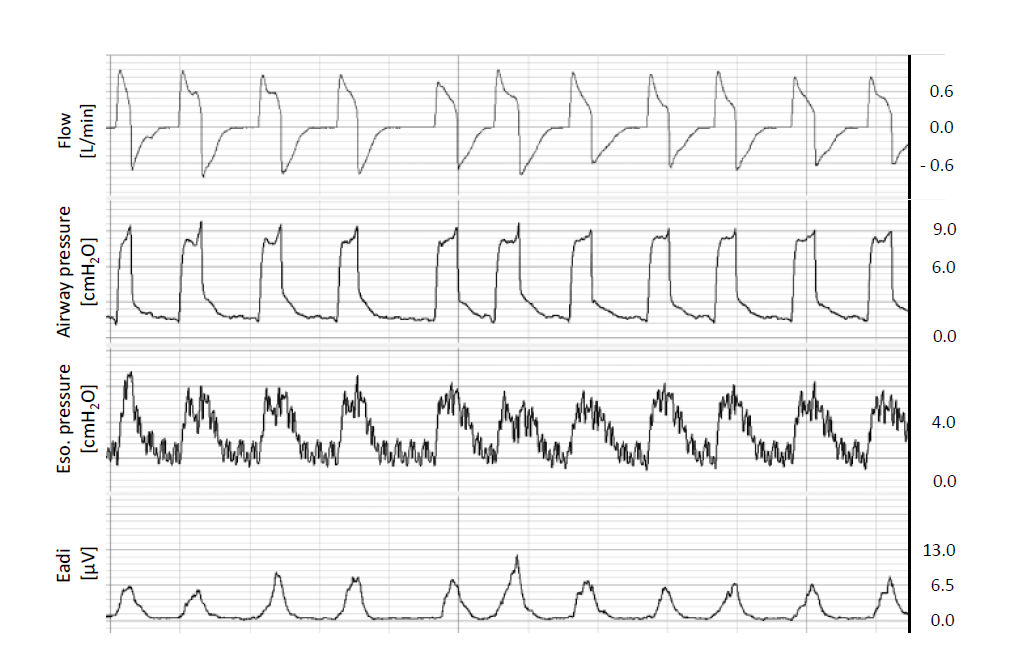
**Figure S2.** Example of over-assist during pressure support ventilation (PSV) indicated by the absence of visible negative esophageal pressure drop. Note that peak electrical activity of the diaphragm (peak Eadi) is on average below 6 microvolts. Eso: esophageal.
